# Supplementary material for: Effects of Music-Based Interventions on Motor and Non-Motor Symptoms in Patients with Parkinson’s Disease: A Systematic Review and Meta-Analysis
Source: Int J Environ Res Public Health. 2023 Jan 6;20(2):1046. doi: 10.3390/ijerph20021046 (PMC9859027; doi:10.3390/ijerph20021046)
Supplement: Supplementary file 1 [file ijerph-20-01046-s001.zip › ijerph-2101011-supplementary.pdf]

### 3.4.1 Cadence

| Sensitivity Analysis | Heterogeneity | Effect size: MD[ 95% CI] |
|---|---|---|
| Bukowska 2016 excluded | $I^2 = 84\%$, $P = 0.002$ | 3.13 [-8.00, 14.26], P = 0.58 |
| Calabrò 2019 excluded | $I^2 = 32\%$, $P = 0.23$ | 6.50 [1.37, 11.62], P = 0.01 |
| Chaiwanichsiri 2011 excluded | $I^2 = 84\%$, $P = 0.002$ | 2.93 [-6.50, 12.35], P = 0.54 |
| Thaut 1996 excluded | $I^2 = 62\%$, $P = 0.07$ | 0.33 [-6.51, 7.17], P = 0.92 |

### 3.5.1 Cognitive flexibility

| Sensitivity Analysis | Heterogeneity | Effect size: MD[ 95% CI] |
|---|---|---|
| Bugos 2021 excluded | $I^2 = 55\%$, $P = 0.14$ | 13.63 [-26.91, 54.17], P = 0.51 |
| Park 2021 excluded | $I^2 = 0\%$, $P = 0.81$ | 39.43 [10.11, 68.75], P = 0.008 |
| Spina 2016 excluded | $I^2 = 75\%$, $P = 0.04$ | 15.33 [-20.38, 51.03], P = 0.40 |

### 3.5.3 Quality of life

| Sensitivity Analysis | Heterogeneity | Effect size: SMD[ 95% CI] |
|---|---|---|
| Bugos 2021 excluded | $I^2 = 91\%$, $P < 0.00001$ | -0.78 [-2.12, 0.56], P = 0.25 |
| Fodor 2021 excluded | $I^2 = 90\%$, $P < 0.00001$ | -0.90 [-2.18, 0.38], P = 0.17 |
| Pacchetti 2000 excluded | $I^2 = 0\%$, $P = 0.64$ | -0.09 [-0.45, 0.27], P = 0.64 |
| Shah 2020 excluded | $I^2 = 91\%$, $P < 0.00001$ | -0.82 [-2.14, 0.49], P = 0.22 |
| Spina 2016 excluded | $I^2 = 90\%$, $P < 0.00001$ | -0.92 [-2.15, 0.31], P = 0.14 |
